# Lacerated Wound of Hip

**Published:** 1881-07

**Authors:** 


### CASE DEPARTMENT.

### COLUMBIA VETERINARY COLLEGE HOSPITAL.

### REPORTS OF CASES UNDER CARE OF FRANK WALTON, D. V. S., HOUSE SURGEON.

I.—Lacerated Wound of Hip.—On February 14th, 1881, a horse was brought to the Columbia Veterinary Hospital suffering from a lacerated wound of the left hind limb, about four inches behind the patella. There was, however, more laceration of the subcutaneous tissue than the external wound would at first indicate. The wound was washed out with carbolic acid solution (1 to 40), and the edges brought together with sutures. The primary inflammation subsided under the influence of aconite and opium. For a few days the animal appeared to be doing well, the wound having closed by first adhesion. On the 17th, however, the leg in the immediate vicinity of the original wound began to swell. The swelling increased rapidly, the sutures were removed, and the wound was reopened. This was followed by a very copious discharge of very offensive pus. The animal had a severe chill, followed by profuse sweating, and the temperature rose to 104° F. Respirations, 20; pulse, 60. The patient seemed to be suffering excruciating pain, which was in part relieved by the free administration of opium. The following day the limb continued rapidly enlarging, although the wound was thoroughly cleansed with carbolic acid solution. The temperature rose to 106° F., respirations to 25, pulse to 70, with rapid loss of strength, the swelling rapidly spreading, and extending up to the abdomen. The animal died from septic poisoning the next morning.

This case is one of especial interest, as it shows how necessary it is, even in small wounds, to establish a free drainage, as many small wounds in the integument are accompanied by extensive laceration of the subcutaneous tissues.

